# Psychometric properties of the Epworth sleepiness scale in Ethiopian university students

**DOI:** 10.1186/s12955-019-1098-9

**Published:** 2019-02-07

**Authors:** Md. Dilshad Manzar, Mohammed Salahuddin, Majed Alamri, Abdulrhman Albougami, Mohammad Yunus Ali Khan, Dejen Nureye, D. Warren Spence, Seithikurippu R. Pandi-Perumal

**Affiliations:** 1 Department of Nursing, College of Applied Medical Sciences, Majmaah University, Majmaah, 11952 Saudi Arabia; 2 Department of Pharmacy, College of Medicine and Health Sciences, Mizan-Tepi University (Mizan Campus), Mizan-Aman, Ethiopia; 3 Biomedical Sciences, College of Medicine and Health Sciences, Mizan-Tepi University (Mizan Campus), Mizan-Aman, Ethiopia; 4 Independent researcher, 652 Dufferin Street, Toronto, ON M6K 2B4 Canada; 5 Somnogen Canada Inc., College Street, Toronto, ON Canada

**Keywords:** Daytime sleepiness, Insomnia, Known-group validity, Floor effect, ESS

## Abstract

**Background:**

Daytime sleepiness is highly prevalent across the globe. The Epworth sleepiness scale (ESS) is the most widely used tool for screening daytime sleepiness. The psychometric properties of the ESS have not been comprehensively examined in African populations.

**Material and methods:**

A cross-sectional design with simple random sampling was used in the present study. The study recruited 600 students from Mizan-Tepi University, Ethiopia, of which 329 (age = 18–28 years and body mass index = 21.19 ± 3.17 kg/m^2^) completed the study. ESS, a semi-structured socio-demographics questionnaire and a clinical interview to diagnose insomnia according to the International Classification of Sleep Disorders were employed.

**Results:**

All except one item of the ESS showed a floor effect, while only one item score showed ceiling effect. However, no ceiling/floor effect was observed in the ESS total score. The Cronbach’s alpha (0.75) and composite reliability (0.75), indicated good internal consistency, while a moderate item-total score correlation (*r* = 0.55–0.67) implied favorable internal homogeneity. The known-group validity was established by significantly higher scores for all the ESS item scores and the ESS total scores among those with symptoms of insomnia than among non-symptomatic students. Fit indices along with the consideration of inter-factor correlation coefficient, measures of item retention favored the unidimensional structure of the ESS.

**Conclusion:**

The ESS has excellent psychometric validity for screening daytime sleepiness in Ethiopian university students.

**Electronic supplementary material:**

The online version of this article (10.1186/s12955-019-1098-9) contains supplementary material, which is available to authorized users.

## Introduction

Daytime sleepiness is characterized by the subjective reporting of disturbances in sustaining alertness while awake, especially associated with quick sleep onset in sedentary situations [1]. It is usually seen in many of the sleep disorders including narcolepsy, hypersomnia, obstructive sleep apnea, delayed sleep phase syndrome, REM sleep behavior disorder, Parkinsonism, sleep-related asthma and insomnia [1]. Daytime sleepiness is a fast-growing sleep-related disorder with reported prevalence rates of 10.4–45.6% across the globe [2–6]. However, the twin problems of frequently underdeveloped sleep health care systems together with the lack of awareness about sleep health matters represent significant barriers to providing patient care in the developing countries of Afro-Asian region [7]. Daytime sleepiness is highly prevalent among university students in Afro-Asian countries including Ethiopia [3, 4]. Moreover, the Ethiopian health care system faces a challenging situation because of limited government resources combined more generally with the psychological and economic difficulties, which populations in developing countries face as part of everyday living [4, 7, 17].

The most objective procedure for assessing daytime sleepiness involves the use of electrophysiological methods, such as those of the Multiple Sleep Latency Test [1]. However, due to the limited health care resources of most healthcare facilities in the developing world, subjective measurements based on comprehensively validated questionnaire tools are the most practical and cost-effective tools for achieving this goal, and thus remain the most important means for screening patients to determine their daytime sleepiness. The Epworth sleepiness scale (ESS) is the most widely used tool for assessing daytime sleepiness [8]. There is considerable evidence showing that the ESS has validated psychometric properties, which make it suitable for screening daytime sleepiness among different populations across the globe [8]. The most important psychometric test properties, such as a test’s construct validity, known-group validity, internal consistency and test-re-test reliability are well established for the ESS in different populations [8]. However, some of the ESS’s validity measures, including the dimensionality and ceiling/floor effects of individual item scores, are not well established [8–15]. Moreover, there is no comprehensively validated tool for screening daytime sleepiness in Africans, including Ethiopians. In view of this deficiency, the present investigators felt that an investigation would be warranted for providing a valid and easy to use questionnaire tool for screening daytime sleepiness in Ethiopians. Therefore, to address the need for establishing the psychometric properties of the ESS in Ethiopian university students, the present study was undertaken. The cosmin guideline and checklists were followed for assessing structural validity (factor analysis), reliability (internal consistency) and known group: discriminative validity in the study population.

## Material and methods

### Participants and study design

A cross-sectional study with a simple random sampling method employing a lottery method was carried out during the April to June, 2018. The study presents analysis of a dataset from a psychological health survey including measures of sleepiness, insomnia and cognition. A total of 600 students were enrolled at Mizan-Tepi University (MTU), Mizan-Aman, Ethiopia. The response rate for the original survey was about 89% with 562 respondents. From this, a dataset of 340 students was randomly selected for this psychometric study. Finally, after removal of person-level missing values (*n* = 11), here we report findings from a sample of 329 students (age = 18–28 years, and body mass index = 21.19 ± 3.17 kg/m^2^). The participating students completed the interviewer-administered original English version of the ESS and then completed a clinical interview and a socio-demographics questionnaire. The English language questionnaires were used because Ethiopia is home to more than 80 languages and students in the university belong to various ethnicities and linguistic subgroups. Due to their diverse backgrounds, the students have differing levels of proficiency in Amharic, the official national language of Ethiopia. Self-reported problems of memory, history of depression, psychosis and those below 13 years of age were excluded. The participants provided informed consent after obtaining a detailed explanation of the purpose and procedures of the study. The research was approved by the institutional Ethical committee, College of Medicine and Health Sciences, Mizan-Tepi University, Ethiopia.

### Measurements

#### The Epworth sleepiness scale

The Epworth Sleepiness Scale (ESS) is an 8-item self-reported questionnaire that is used to assess the self-reported level of daytime sleepiness [16]. These 8 items have a four-point scale, where, ‘0’ indicates ‘would never nod off’, while ‘3’ indicates a strong chance of nodding off, scale questions which are applied to 8 different situations encountered in daily life [16]. All the individual item scores are added to generate the ESS total score that ranges between 0 and 24. Higher ESS total scores indicate a progressively greater degree of daytime sleepiness in the respondent [16].

#### Socio-demographics questionnaire

The participants filled out an interviewer-administered semi-structured socio-demographics questionnaire with 10-items; 3-open-ended and 7-closed-ended. These items collected responses regarding age, gender, religion, parent’s marital status, physical activity, years of university education, attendance in lectures and practical classes, and presence of chronic conditions. Height and weight were measured to determine body mass index.

#### Clinical interview for symptoms of insomnia

The participants were interviewed by an experienced sleep researcher blinded to their ESS scores to screen for the presence of the symptoms of insomnia. Insomnia was defined according to criteria listed in the International Classification of Sleep Disorders (ICSD-2) [1, 17, 18]. The diagnosis was based on (i) reporting insufficient sleep occurring almost nightly, (ii) complaints of restlessness after sleep, (iii) mild to moderate levels of socio-occupational impairment, (iv) complaints of problems such as anxiety, daytime fatigue, irritability, and tiredness. The presence of insomnia symptoms was based on whether respondents had either of the first two conditions together with the presence of the last two i.e. (iii) and (iv) [1]. Insomnia patients usually have increased levels of daytime sleepiness; therefore, difference in the ESS scores between two groups of students, with complaints of insomnia and other without insomnia (normal).

### Data analysis

Data analysis using SPSS 22.0 with Amos was carried out. Mean ± standard deviations (SD), frequency, and percentage were used to describe participants’ characteristics as well as the distributions of ESS scores. The Cronbach’s alpha test assessed the internal consistency of the data; Spearman’s correlation test measured Item-total correlation & inter-item correlations, an measure for assessing the internal homogeneity of the data. Independent t-tests and Mann Whitney tests were used to evaluate the known-group validity - discriminative validity of the ESS among the Ethiopian students.

**Fig. 1:**
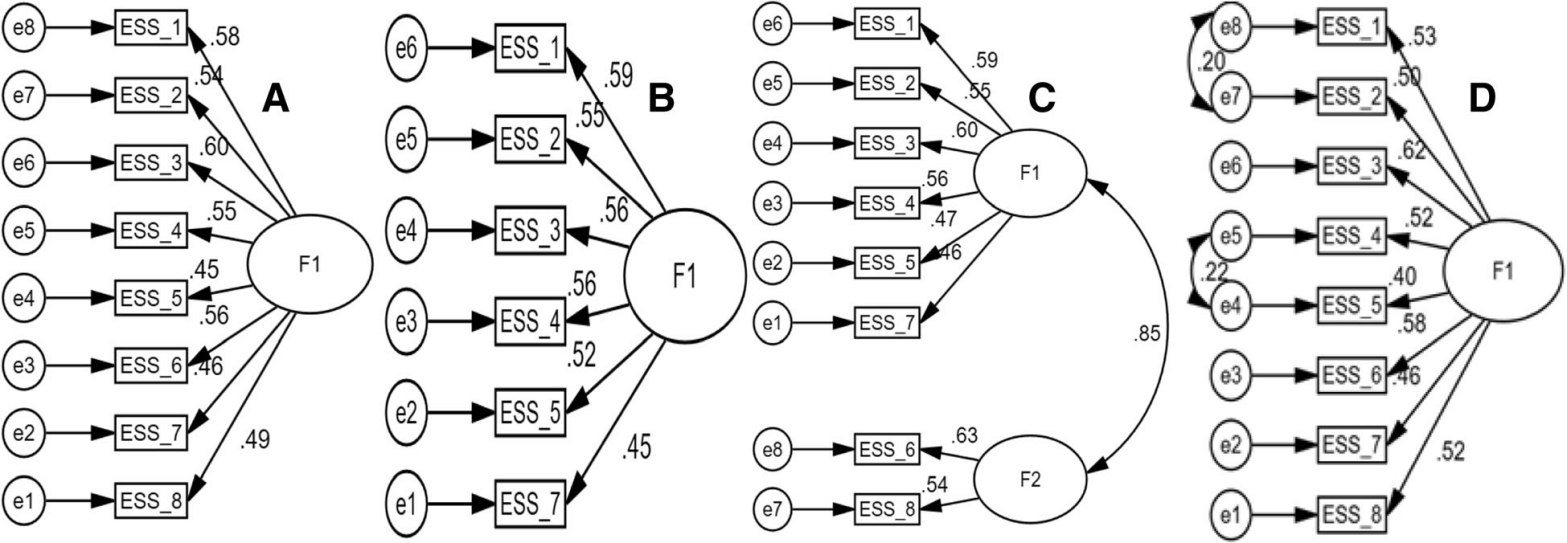
Confirmatory factor analysis of the Epworth Sleepiness Scale (ESS) scores in Ethiopian university students. ESS_1 to ESS_8: Items of the ESS; A: 1-Factor model, B: 1-Factor model (Smith et al. 2008), C: 2-Factor model (Gelaye et al. 2014), D: 1-Factor model with incorporation of modification indices (co-varying error terms). All coefficients are standardized. *Ovals* latent variables, *rectangles* measured variables, *circles* error terms, *single-headed arrows* between *ovals* and *rectangles* factor loadings, *single-headed arrows* between *circles* and *rectangles* error terms. Smith SS, Oei TP, Douglas JA, Brown I, Jorgensen G, Andrews J. Confirmatory factor analysis of the Epworth Sleepiness Scale (ESS) in patients with obstructive sleep apnoea. Sleep medicine. 2008 Oct 1;9(7):739–44. Gelaye B, Lohsoonthorn V, Lertmeharit S, Pensuksan WC, Sanchez SE, Lemma S, Berhane Y, Zhu X, Vélez JC, Barbosa C, Anderade A. Construct validity and factor structure of the pittsburgh sleep quality index and epworth sleepiness scale in a multi-national study of African, South East Asian and South American college students. PloS one. SSS 31;9(12):e116383

Confirmatory factor analysis (CFA) was performed using the maximum likelihood extraction with bootstrap; to manage multivariate non-normality. Standardized estimates of factor loadings by each ESS item scores on their factor(s) were determined. Furthermore modification indices, co-varying error terms, was assessed to explore model improvement for better fit. The dimensionality of the ESS is debatable; therefore, a CFA was performed on the previously reported factor structures of the ESS [8–14, 19]. CFA was used to screen four models; A: 1-Factor model, B: 1-Factor model (a shortened ESS scale with 6-items) [11], C: 2-Factor model [13], D: 1-Factor model with incorporation of modification indices (co-varying error terms) (Table 6, Fig. 1). The previously accepted practice of using multiple fit indices from different categories was employed [19, 20, 27]. These included absolute fit indices including the goodness of fit index (GFI), root mean square residual (RMR), and χ^**2**^, relative fit index-incremental fit index (IFI) and non-centrality indices such as the root mean square error of approximation (RMSEA) and comparative fit index (CFI) [20]. A non-significant χ^**2**^ value indicated an absolute fit between the observed and expected model values. The threshold of RMSEA (≤ .08), RMR (≤ 0.05) and χ^**2**^/df (≤ 2) indicated that an excellent fit existed [21]. A value greater than 0.95 for CFI, GFI and IFI implied excellent fit [21].

## Results

### Participants’ characteristics

Table 1 describes the socio-demographics of the participants. The mean ages and ESS scores of the Ethiopian university students were 20.96 ± 1.74 years and 6.83 ± 4.65, respectively (Table 1). The majority of the participants (67.8%) were males (Table 1). More than 1/10th of the study populations (10.6%) reported having chronic conditions such as Acquired immune deficiency syndrome, hepatitis-A/B, hypertension, diabetes mellitus, tuberculosis and others (Table 1). Most of the students (75.7%) reported physical activity such as running, walking, and other exercises (Table 1). The prevalence of symptoms of insomnia was 44.7% (Table 1).

**Table 1:** Socio-demographics of Ethiopian university students (*n* = 329) participating in the study

Characteristics	Mean ± SD/ Frequency
Age (yr)	20.96 ± 1.74
BMI (Kg/m^2^)	21.19 ± 3.17
Gender	
Male	223(67.8%)
Female	88(26.7%)
Unreported	18 (5.5%)
Years of university education	
1 year	98 (29.8%)
2 year	122 (37.1%)
3 Year	63 (19.1%)
4 Year	25 (7.6%)
5 Year	4 (1.2%)
Unreported	17 (5.2%)
Religion	
Orthodox	142 (43.2%)
Protestants	40 (12.2%)
Islam	120 (36.5)
Others	4 (1.2%)
Unreported	23 (7.0%)
Attendance (%)	
51–60	3 (0.9%)
61–70	2 (0.6)
71–80	16 (4.9%)
81–90	31 (9.4%)
91–100	187 (56.8%)
Unreported	90 (27.4%)
Chronic conditions	
None	195 (59.3%)
AIDS	8 (2.4%)
Hepatitis-A	3 (0.9%)
Hepatitis-B	1 (0.3%)
Hypertension	1 (0.3%)
Diabetes Mellitus	3 (0.9%)
Tuberculosis	5 (1.5%)
Others	14 (4.3%)
Unreported	99 (30.1%)
Parents	
Single	120 (36.5%)
Divorced	9 (2.7%)
Widowed	14 (4.3%)
Double	133 (40.4%)
Unreported	53 (16.1%)
Physical Activity	
No	62 (18.8%)
Yes	249 (75.7%)
Unreported	18 (5.5%)
ESS	6.83 ± 4.65
Symptoms of Primary insomnia	
Yes	147 (44.7%)
No	182 (55.3%)

*SD*: Standard deviation, *BMI*: Body mass index; *ESS*: Epworth Sleepiness Scale

### Item analysis

Table 2 shows the descriptive analysis of the ESS scores among the participating students. All the items showed the floor effect as indicated by the fact that more than 15% of the participants endorsed the lowest score [17, 22]. With the exception of ESS-5, none of the items showed evidence of a ceiling effect, as indicated by the fact that less than 15% of the respondents endorsed the highest level of agreement for the item. [17, 22]. There were no issues of the floor effect or the ceiling effect for the ESS total score with only 11.6% respondents reporting lowest score of 0, and none reported the highest score of 24, with a range of 0–21. [17, 22]. All the ESS item scores (except ESS item-5 score) and the ESS total score were skewed as evidenced by the values of the Z-score of the skewness (≥3.29) (Table 2). However, there was issue of kurtosis for three of the ESS item scores, ESS item-2, ESS item-5 and ESS item-6, all of these three items had Z-score of the kurtosis (≥3.29) (Table 2).

**Table 2:** Descriptive statistics of the Epworth Sleepiness Scale (ESS) scores in Ethiopian university students

Epworth Sleepiness Scale items	Mean ± SD	Skewness		Kurtosis		Item scores			
		Statistics (SE)	Z-Score	Statistics (SE)	Z-Score	0 Frequency (%)	1 Frequency (%)	2 Frequency (%)	3 Frequency (%)
Sitting and Reading	0.94 ± 0.90	.59 (.13)	4.37	−.59 (.27)	−2.19	125 (38.0%)	117 (35.6%)	68 (20.7%)	19 (5.8%)
Watching TV	0.59 ± 0.91	1.42(.13)	10.58	.94(.27)	3.49	209 (63.5%)	67 (20.4%)	32 (9.7%)	21 (6.4%)
Sitting inactive in a public place (i.e., a theatre)	0.78 ± 0.90	.93(.13)	6.91	−.08(.27)	−.29	159 (48.3%)	103 (31.3%)	48 (14.6%)	19 (5.8%)
As a car passenger for an hour without a break	0.97 ± 1.05	.73(.13)	5.40	−.76(.27)	−2.82	146 (44.4%)	90 (27.4%)	51 (15.5%)	42 (12.8%)
Lying down to rest in the afternoon	1.48 ± 1.12	.01(.13)	.04	−1.37(.27)	−5.12	87 (26.4%)	77 (23.4%)	86 (26.1%)	79 (24.0%)
Sitting and talking to someone	0.56 ± 0.81	1.35(.13)	10.01	.97(.27)	3.62	202 (61.4%)	81 (24.6%)	35 (10.6%)	11 (3.3%)
Sitting quietly after lunch without alcohol	0.74 ± 0.98	1.10(.13)	8.19	.01(.27)	.02	184 (55.9%)	76 (23.1%)	41 (12.5%)	28 (8.5%)
In a car, while stopping for a few minutes in traffic	0.78 ± 0.97	1.03(.13)	7.67	−.04(.27)	−.13	168 (51.1%)	93 (28.3%)	39 (11.9%)	29 (8.8%)
ESS total score		.46(.13)	3.44	−.07(.27)	−.26				

*SD*: Standard deviation; *SE*: Standard error

### Internal consistency and homogeneity

The Cronbach’s alpha for the ESS was 0.75, while Cronbach’s Alpha if item deleted ranged from 0.72–0.74 (Table 3). The Spearman’s correlation coefficient for the item-total score ranged between 0.55 and 0.67 (*p* < 0.01) (Table 3). The inter-item Spearman’s correlation coefficient ranged between 0.12 and 0.40 (*p* < 0.01 or *p* < 0.05) (Table 4).

**Table 3:** Internal consistency of the Epworth Sleepiness Scale (ESS) scores in Ethiopian university students

	Item-Total Correlation	Cronbach’s Alpha if Item Deleted	Communality
Sitting and Reading	.61^*^	.72	.42
Watching TV	.59^*^	.72	.39
Sitting inactive in a public place (i.e., a theatre)	.67^*^	.72	.45
As a car passenger for an hour without a break	.64^*^	.72	.40
Lying down to rest in the afternoon	.60^*^	.74	.29
Sitting and talking to someone	.55^*^	.72	.40
Sitting quietly after lunch without alcohol	.57^*^	.74	.30
In a car, while stopping for a few minutes in traffic	.55^*^	.73	.33

^*^p < 0.01

**Table 4:** Inter-item Correlation matrix of the Epworth Sleepiness Scale (ESS) scores in Ethiopian university students

	ESS item-1	ESS item-2	ESS item-3	ESS item-4	ESS item-5	ESS item-6	ESS item-7	ESS item-8
ESS item-1		.40^**^	.31^**^	.32^**^	.27^**^	.28^**^	.24^**^	.26^**^
ESS item-2			.32^**^	.23^**^	.25^**^	.34^**^	.28^**^	.23^**^
ESS item-3				.38^**^	.26^**^	.36^**^	.36^**^	.36^**^
ESS item-4					.36^**^	.26^**^	.26^**^	.32^**^
ESS item-5						.14^*^	.25^**^	.12^*^
ESS item-6							.29^**^	.35^**^
ESS item-7								.30^**^
ESS item-8								

^*^ *p* < 0.05; ^**^ *p* < 0.01

### Known-group validity: Discriminative validity

The groups of Ethiopian university students identified as normal and those with symptoms of insomnia as determined by clinical interview differed significantly across the ESS total score and the ESS item scores (*p* ≤ .001) (Table 5).

**Table 5:** Discriminative validity: Comparison of the of the Epworth Sleepiness Scale (ESS) scores between normal and primary insomnia groups as determined by clinical interview in Ethiopian university students

Items of the ESS	Mean		*p*-value
	Normal sleepers (*n* = 182)	Primary Insomnia (*n* = 147)	
Sitting and Reading	0.79 ± 0.89	1.14 ± 0.89	<.001
Watching TV	0.41 ± 0.84	0.82 ± 0.94	<.001
Sitting inactive in a public place (i.e., a theatre)	0.50 ± 0.77	1.12 ± 0.94	<.001
As a car passenger for an hour without a break	0.63 ± 0.86	1.39 ± 1.12	<.001
Lying down to rest in the afternoon	1.18 ± 1.14	1.85 ± 0.99	<.001
Sitting and talking to someone	0.34 ± 0.64	0.83 ± 0.92	<.001
Sitting quietly after lunch without alcohol	0.45 ± 0.80	1.09 ± 1.06	. < .001
In a car, while stopping for a few minutes in traffic	0.64 ± 0.92	0.97 ± 0.97	.001
ESS total score*	4.92 ± 3.99	9.20 ± 4.31	<.001

*Mean ± SD, Independent t-test was used for the ESS total score and Mann Whitney U test was applied for item scores

### Factor analysis

#### Measures indicating sample adequacy, suitability and factorability

All the diagonal elements of the anti-image matrix of the correlations were greater than 0.5 with a range between 0.79 and 0.87, and the off-diagonal elements, i.e., partial correlations between the ESS item scores, were small as required for the factor analysis [25]. The Kaiser-Meyer-Olkin test of sampling adequacy (0.83) implied that the sample had a meritorious degree of common variance [25]. The significant value for Bartlett’s test of sphericity suggested that the ESS items’ observed correlation matrix was statistically different from a singular matrix and established the existence of linear combinations among the ESS item scores [25]. The determinant of the correlation matrix (0.25) support the factorability of the ESS item scores because it indicated the absence of singularity as well as the absence of multicollinearity [25]. The communality values were in the range between 0.29 and 0.45 (Table 3), thus indicating that a satisfactory level of variance was explained by the common factors; therefore all the ESS items were retained for factor analysis [26–28]. Thirteen of the inter-item correlations for the ESS scores were greater than 0.3 (Table 4), implying factorability of the correlation matrix and the absence of the multicollinearity [29].

#### Confirmatory factor analysis

Table 6 shows the fit statistics of the four factor structures of the ESS scores in the study population. Model-D; a 1-Factor model with incorporation of modification indices (co-varying error terms) was found to have absolute fit to the data i.e. a non-significant χ^2^ *p*-value, and excellent fit as indicated by the values for RMSEA (0.03(0.00–0.06)), GFI (0.98), IFI (0.99), CFI (0.99), RMR (0.03) and χ^2^/df (1.26) (Table 6) [31]. Model-D showed factor loadings in the range of 0.40 and 0.63 (Fig. 1), suggesting that there was fair to a good range of overlapping variance, i.e., correlations between the ESS item scores and its factor [30].

**Table 6:** Fit statistics of the Epworth Sleepiness Scale (ESS) scores in Ethiopian university students

Models	GFI	IFI	CFI	RMR	RMSEA	χ^2^	df	*p*	χ^2^/df
A	.97	.94	.94	.04	.06(.04–.09)	45.31	20	.001	2.27
B	.98	.96	.96	.04	.06(.02–.10)	20.26	9	.016	2.25
C	.97	.95	.95	.04	.06(.04–.08)	41.25	19	.002	2.17
D	.98	.99	.99	.03	.03(.00–.06)	22.61	18	.206	1.26

A: 1-Factor model, B: 1-Factor model (Smith et al. 2008), C: 2-Factor model (Gelaye et al. 2014),

D: 1-Factor model with incorporation of modification indices (co-varying error terms)

*GFI*: Goodness of fit index, *IFI*: Incremental Fit Index, *CFI*: Comparative Fit Index, *RMR*: root mean square residual, *RMSEA*: root mean square error of approximation

Smith SS, Oei TP, Douglas JA, Brown I, Jorgensen G, Andrews J. Confirmatory factor analysis of the Epworth Sleepiness Scale (ESS) in patients with obstructive sleep apnoea. Sleep medicine. 2008 Oct 1;9(7):739–44

Gelaye B, Lohsoonthorn V, Lertmeharit S, Pensuksan WC, Sanchez SE, Lemma S, Berhane Y, Zhu X, Vélez JC, Barbosa C, Anderade A. Construct validity and factor structure of the pittsburgh sleep quality index and epworth sleepiness scale in a multi-national study of African, South East Asian and South American college students. PloS one. 2014 Dec 31;9(12):e116383

## Discussion

This is the first comprehensive psychometric validation investigation of the original English version of the ESS in the African population in general and among Ethiopian university students in particular. The study assessed item analysis-the ceiling effect and the floor effect, internal consistency, internal homogeneity, known-group validity and factor analysis of the ESS. In accordance with Cosmin checklist, the study got a ‘very good’ score for methodological quality in assessment of internal consistency using Cronbach’s alpha, structural validity employing both EFA and CFA without methodological discrepancies and appropriate statistics for known-group: discriminative validity.

Despite the comparability of psychometric properties following administration of the ESS among the presently studied sample and world samples, most of the ESS item scores showed the floor effect and but only one of them showed the ceiling effect. Moreover, the ESS total score did not have either of these effects [17, 22]. The floor effect and the ceiling effects on individual ESS items is an under-investigated measure [8]. Similar to our findings in this Ethiopian student population, Hagell and Broman 2007 reported no major issues of the floor and ceiling effects for the ESS total score in a study sample of Swedish Parkinson’s patients [10]. The ceiling/floor effects may affect the responsiveness of the ESS item scores [22]. Indeed, previous studies have expressed reservations about the responsiveness of the ESS in a normative population as well as in elderly [23, 24]. One explanation for the presence of the floor effect in all but one of the ESS item scores in this study may be that the study population was non-clinical. This reasoning is supported by the finding of a floor effect for all the item scores of the Hospital anxiety and depression scale reported when tested among a general elderly Swedish population [32]. In summary, the absence of the ceiling/floor effect for the ESS total score does support its applicability in the Ethiopian students [32].

The internal consistency as assessed by the Cronbach’s alpha was good in the study population [33]. The composite reliability of the unidimensional Model-D of the ESS was 0.75, which is reflective of and therefore reinforces its internal consistency in Ethiopian university students. Previous studies have reported the Cronbach’s alpha for the ESS in the range of 0.73–0.86 [8]. In 1992, John reported a slightly lower value of the Cronbach’s alpha of 0.72 among medical students [34]. The item-total correlations for the ESS scores indicate a moderate level of internal homogeneity for the scale in this population. In 2014, Sargento and co-workers reported similar values in the range of 0.43–0.73 among Portuguese adults [9]. The inter-item correlations indicated a weak to moderate level of internal homogeneity. However, this measure of internal validity was relatively higher than that reported for the sample of Portuguese adults (r = 0.05–0.47) [9]. Similarly, Baumgartel and colleagues reported a range of r = 0.07 to r = 0.46 for the inter-item correlations for the ESS scores among an American obstetric population [12].

The diagnostic known-group validity or discriminative validity was strongly supported by the significantly higher values for all the ESS item scores as well as the ESS total score among the subset of Ethiopian students with symptoms of insomnia when compared to those who did not have insomnia symptoms. Insomnia patients usually have increased levels of daytime sleepiness; therefore, the higher values for the ESS item as well as the ESS total score establish the validity of the scale in Ethiopian university students [1]. In comparison with other sleep questionnaires validated among Ethiopians, the ESS is on par with the Leeds sleep evaluation questionnaire and slightly better than the Pittsburgh sleep quality index [17, 18]. For the Leeds sleep evaluation questionnaire, all item scores, as well as the total scores, differed significantly among known-groups, while for the Pittsburgh sleep quality index, two item scores did not differ among known groups [17, 18].

Though, model fit indices showed little difference between the four models evaluated in the study population. The validity of model-C, a 2-Factor model was not favored because, the inter-factor correlation was 0.85, which suggested poor divergent validity of two factors (Table 6, Fig. 1) [19]. Though, performance of model-B according to fit indices was satisfactory, but its application is not recommended because it has 2 items less than the original ESS [11, 16]. This might lead to loss of important information about respondent’s sleepiness [16]. Furthermore, there was no indication to delete these two items in the study population as implied by values of (i) Cronbach’s alpha if item deleted, (ii) Communality and (iii) factor loadings (Table 3, Table 6, and Fig. 1) [19, 30]. Model-D had slightly better fit compared to model-A considering values for GFI, IFI, CFI, RMR and RMSEA. However, only model-D had absolute fit as indicated by a non-significant χ^2^ test and it also had least value for χ^2^/df (Table 6, Fig. 1). Therefore, the validity of Model-D, in the study population is favored. This is similar to the structural validity reports of the ESS in previous studies of Portuguese adults and Swedish Parkinson’s patients [9, 10]. However, the unidimensionality of the ESS has been debatable because of the disparate findings in different populations [8]. Smith et al. 2008 validated a slightly re-specified 1-Factor model (sans item-6 and item-8) using CFA among Australian obstructive sleep apnea patients [11]. Their model was not found to be valid in our study population. Some studies reported 2-Factor models in different Population [12–14]. However, unlike our study, Baumgartel et al. 2013 did not implement the modification indices and did not report values for any of the relative fit indices; therefore, a direct comparison is difficult [12]. In 2018, Pilcher and co-workers did not perform CFA [12]; this is contrary to recommended practices, especially when the dimensionality of the scale is debatable [19]. In 2014, Gelaye and co-workers did not incorporate modification indices into their analysis and reported values for only two categories of the fit indices, i.e., non-centrality fit indices (RMSEA and CFI) and a relative fit index (TLI) [13]. The findings of Nguyen et al. (2006) supported the inference of a 3-Factor model in Canadian patients who had complaints of snoring [15]. However, it is difficult to infer the applicability of their 3-Factor model because the study gives little detail about factor analysis, and did not employ CFA [15].

The limitations of the present study included the fact that it did not carry out assessments of the concurrent validity, convergent validity, test-re-test reliability, and had a small number of female participants, thus limiting its generalizability primarily to males. The multiple sleep latency tests were not performed for the assessment of the concurrent validity because polysomnographic testing was not available. A number of the female students did not complete the clinical interview, resulting in a somewhat unbalanced gender proportion in the final study sample. Future studies should employ Rasch analysis as it is a robust measure of item performance. Though, this was a single centric study, but MTU students participating in this study belonged to almost all regions of Ethiopia, therefore the results may be generalizable to students of other universities with some limitations.

## Conclusion

Taking all the limitations into consideration, nonetheless, the general overall favorable psychometric properties support the validity of the ESS for the screening of daytime sleepiness among Ethiopian university students.

## Additional file

Additional file 1: Supplementary data. (SAV 10 kb)

## Abbreviations

CFA: Confirmatory factor analysis
CFI: Comparative Fit Index
EFA: Exploratory factor analysis
ESS: Epworth sleepiness scale
GFI: Goodness of fit index
ICSD: International Classification of Sleep Disorders
IFI: Incremental Fit Index
RMR: Root mean square residual
RMSEA: Root mean square error of approximation
SD: Standard deviations

## References

[1] AASM (2001). International classification of sleep disorders, revised: diagnostic and coding manual. Chicago.

[2] Hayley AC, Williams LJ, Kennedy GA, Berk M, Brennan SL, Pasco JA (2014). Prevalence of excessive daytime sleepiness in a sample of the Australian adult population. Sleep Med.

[3] Kaur G, Singh A (2017). Excessive daytime sleepiness and its pattern among Indian college students. Sleep Med.

[4] Robinson D, Gelaye B, Tadesse MG, Williams MA, Lemma S, Berhane Y (2013). Daytime sleepiness, circadian preference, caffeine consumption and Khat use among college students in Ethiopia. J Sleep Disord Treat Care.

[5] Langberg JM, Molitor SJ, Oddo LE, Eadeh HM, Dvorsky MR, Becker SP. Prevalence, patterns, and predictors of sleep problems and daytime sleepiness in young adolescents with ADHD. J Atten Disord. 2017 Jan 1:1087054717690810. 10.1177/1087054717690810 28162039

[6] Hein M, Lanquart JP, Loas G, Hubain P, Linkowski P (2017). Prevalence and risk factors of excessive daytime sleepiness in insomnia sufferers: a study with 1311 individuals. J Psychosom Res.

[7] Manzar MD, Salahuddin M, Maru TT, Dadi TL, Abiche MG, Abateneh DD, Pandi-Perumal SR, Bahammam AS (2017). Sleep correlates of substance use in community-dwelling Ethiopian adults. Sleep and Breathing..

[8] Kendzerska TB, Smith PM, Brignardello-Petersen R, Leung RS, Tomlinson GA (2014). Evaluation of the measurement properties of the Epworth sleepiness scale: a systematic review. Sleep Med Rev.

[9] Sargento P, Perea V, Ladera V, Lopes P, Oliveira J (2015). The Epworth sleepiness scale in Portuguese adults: from classical measurement theory to Rasch model analysis. Sleep and Breathing.

[10] Hagell P, Broman JE (2007). Measurement properties and hierarchical item structure of the Epworth sleepiness scale in Parkinson's disease. J Sleep Res.

[11] Smith SS, Oei TP, Douglas JA, Brown I, Jorgensen G, Andrews J (2008). Confirmatory factor analysis of the Epworth sleepiness scale (ESS) in patients with obstructive sleep apnoea. Sleep Med.

[12] Baumgartel KL, Terhorst L, Conley YP, Roberts JM (2013). Psychometric evaluation of the Epworth sleepiness scale in an obstetric population. Sleep Med.

[13] Gelaye B, Lohsoonthorn V, Lertmeharit S, Pensuksan WC, Sanchez SE, Lemma S, Berhane Y, Zhu X, Vélez JC, Barbosa C, Anderade A (2014). Construct validity and factor structure of the Pittsburgh sleep quality index and Epworth sleepiness scale in a multi-national study of African, south east Asian and south American college students. PLoS One.

[14] Pilcher JJ, Switzer FS, Munc A, Donnelly J, Jellen JC, Lamm C (2018). Psychometric properties of the Epworth sleepiness scale: a factor analysis and item-response theory approach. Chronobiol Int.

[15] Nguyen AT, Baltzan MA, Small D, Wolkove N, Guillon S, Palayew M (2006). Clinical reproducibility of the Epworth sleepiness scale. J Clin Sleep Med.

[16] Johns MW (1991). A new method for measuring daytime sleepiness: the Epworth sleepiness scale. Sleep.

[17] Salahuddin M, Maru TT, Kumalo A, Pandi-Perumal SR, Bahammam AS, Manzar MD (2017). Validation of the Pittsburgh sleep quality index in community dwelling Ethiopian adults. Health Qual Life Outcomes.

[18] Manzar MD, Salahuddin M, Maru TT, Alghadir A, Anwer S, Bahammam AS, Pandi-Perumal SR (2018). Validation of the adapted Leeds sleep evaluation questionnaire in Ethiopian university students. Health Qual Life Outcomes.

[19] Manzar MD, BaHammam AS, Hameed UA, Spence DW, Pandi-Perumal SR, Moscovitch A, Streiner DL (2018). Dimensionality of the Pittsburgh sleep quality index: a systematic review. Health Qual Life Outcomes.

[20] Jaccard J, Wan C: LISREL Approaches to Interaction Effects in Multiple Regression. Vol. 114 (Quantitative Applications in the Social Sciences), 1^st^ edition: SAGE PublicationsInc; 1996, 112 pages.

[21] Browne MW, Cudeck R, Bollen& KA, Long JS (1993). Alternative ways of assessing model fit. Testing structural equation models.

[22] Lim CR, Harris K, Dawson J, Beard DJ, Fitzpatrick R, Price AJ (2015). Floor and ceiling effects in the OHS: an analysis of the NHS PROMs data set. BMJ Open.

[23] Onen F, Onen SH (2010). Fundamentals of quality of life and daytime sleepiness measurements in older sleep apnea patients. Sleep Med.

[24] Sanford SD, Lichstein KL, Durrence HH (2006). The influence of age, gender, ethnicity, and insomnia on Epworth sleepiness scores: a normative US population. Sleep Med.

[25] Field A. (2017) Discovering statistics using IBM SPSS statistics. SAGE Publications Limited, 5^th^ edition, ISBN-13: 978–9351500827, 1324 pages.

[26] Child, D. (2006). The essentials of factor analysis. 3rd edition. New York, NY: Bloomsbury AcademicISBN-13: 978–0826480002, 192 pages.

[27] Manzar MD, Zannat W, Moiz JA, Spence DW, Pandi-Perumal SR, Bahammam AS (2016). Factor scoring models of the Pittsburgh sleep quality index: a comparative confirmatory factor analysis. Biol Rhythm Res.

[28] Manzar MD, Zannat W, Hussain ME, Pandi-Perumal SR, Bahammam AS, Barakat D, Ojike NI, Olaish A, Spence DW (2016). Dimensionality of the Pittsburgh sleep quality index in the collegiate young adults. Springerplus.

[29] Tabachnick BG, Fidell LS (2007). Using multivariate statistics.

[30] Comrey, A. L., & Lee, H. B. (1992). A first course in factor analysis, 2nd edition, Hillsdale, NJ: Lawrence Erlbaum Associates.ISBN: 0805810625,430 pp.

[31] Hu LT, Bentler PM. Cutoff criteria for fit indexes in covariance structure analysis: conventional criteria versus new alternatives.Struct Equ Model Multidiscip J Volume 6, 1999 - Issue 1Volume 6, 1999, Issue 1. Pages 1–55.

[32] Djukanovic I, Carlsson J, Årestedt K (2017). Is the hospital anxiety and depression scale (HADS) a valid measure in a general population 65–80 years old? A psychometric evaluation study. Health Qual Life Outcomes.

[33] DeVellis, R.F. (2012). Scale development: theory and applications. Los Angeles: 3^rd^ edition, Sage. pp. 109–110.ISBN: 9781412980449.

[34] Johns MW (1992). Reliability and factor analysis of the Epworth sleepiness scale. Sleep.

